# Cognitive assessment during the phases of a spontaneous migraine: a prospective cohort study

**DOI:** 10.1007/s10072-024-07520-w

**Published:** 2024-04-12

**Authors:** Jason C. Ray, David Darby, Helmut Butzkueven, Manjit S. Matharu, Elspeth J. Hutton

**Affiliations:** 1 Department of Neurology, Alfred Hospital, Commercial Road, Melbourne, VIC 3004 Australia; 2 Department of Neuroscience, Central Clinical School, Monash University, 99 Commercial Road, Melbourne, VIC 3004 Australia; 3 Department of Neurology, Austin Health, 145 Studley Road, Heidelberg, 3084 Australia; 4 Headache and Facial Pain Group, University College London (UCL) Queen Square Institute of Neurology, The National Hospital for Neurology and Neurosurgery, Queen Square, London, WC1N 3BG UK

**Keywords:** Migraine, Cognition, Cognitive impairment, Headache disorders

## Abstract

**Introduction:**

Cognitive symptoms are reported commonly throughout all phases of a migraine; however, there is a paucity of objective cognitive profiling. Previous studies have been limited by practice effect, and variable populations.

**Methods:**

Participants completed 1 month of daily testing with a computerised cognitive battery involving a simple reaction (SRT), choice reaction (CRT) and a working memory test (WM). Results were correlated with their diary to identify interictal scores, and scores during each phase of a migraine, and non-migraine headache days.

**Results:**

A total of 16 patients with episodic migraine participated. During the headache phase of a migraine, responses to SRT, CRT and WM tasks were significantly slower and less accurate than interictally. During the postdrome, WM task performance was slower and less accurate. Non-migraine headache days were not associated with significant change.

**Conclusion:**

The headache and postdromal phase of a migraine day was associated with objective evidence of cognitive dysfunction in patients with episodic migraine.

## Introduction

Cognitive dysfunction is the second most disabling symptom of a migraine attack, and is likely to be responsible for some of the significant economic burden of migraine [1]. Cognitive dysfunction is the most frequent symptom complex in the prodromal phase [2]. It persists throughout the headache phase, during which up to 90% of patients report the inability to concentrate, and the postdromal phase, in which 40% of patients report impaired concentration [2, 3].

Use of neuropsychological tests to assess cognitive effects of migraine is limited by variance in healthy adults with higher cognitive baselines, practice effects of repeat testing, and the burden of repeat testing for clinicians and patients [4–6]. Several previous studies have attempted to assess cognition during a migraine attack, and have variably found transient declines in processing speed and working memory [7–9]. The goal of this study is to use a previously validated and very short set of computerised cognitive tests that patients can complete at home to assess the impact of cognitive dysfunction during each phase of a migraine, and a non-migrainous headache day in comparison to the interictal baseline.

## Methodology

We undertook a single-centre prospective cohort study in patients who met the International Classification of Headache Disorders, third edition (ICHD-3) criteria for episodic migraine. Participants who were on stable preventative treatment for their migraine, did not take opiates for acute treatment and had no confounding medical or psychiatric comorbidity were eligible for the study.

Participants were educated on identifying migraine days and non-migraine headache days by a headache specialist, and then, following a practice session, asked to complete 1 month of daily home testing while keeping a headache diary. Participants had a computerised cognitive battery (“MSReactor”), and were asked to perform a test at the same time daily for the study duration. Participants were allowed to continue a stable dose of their usual acute and preventative migraine treatment throughout the study period.

The MSReactor test battery is accessible to participants via the internet, and consists of three simple graphical game-like tests including a psychomotor/simple reaction test (SRT), a visual attention choice reaction test (CRT) and a working memory one-back test (WM) [10]. The test battery that has previously been utilised in several neurological disorders, including migraine, has demonstrated correlation with more extensive cognitive tests, and demonstrates limited practice effects, reaching a stable baseline within three repetitions [10–12].

Practise, or learning effect, is a significant consideration in the concurrent application of test batteries. The learning effect and test–retest reliability of the MSReactor battery has been determined previously [10]. The learning effect of this battery stabilises within three repetitions. To account for the impact of learning effect on the study, the first five repetitions for each participant were considered practices, and not included in the analysis.

The impact of migraine state on test results was measured by reaction speed (milliseconds) and test accuracy (percentage of correct responses in each trial). The first 5 days were not included in the analysis to allow stabilisation of practice effect. Interictal tests were defined as tests that were completed on days at least 2 days separate from a recorded migraine or headache, and these results were averaged to provide a patient’s interictal baseline.

Where a patient had more than one qualifying event, the first qualifying migraine or non-migraine headache day was chosen for analysis. The prodrome and postdrome phase in the study was defined as the day of testing preceding or following the headache phase on a migraine day, as marked in the patient diary.

Statistical analysis was performed using SPSS v28.0. Population characteristics were summarised with descriptive statistics. A Wilcoxon signed rank test was used to analyse non-normally distributed paired samples. Test results were considered significant when *p* < 0.05. This study received institutional review board approval (HREC 153/21).

## Results

A total of 16 patients with episodic migraine were enrolled in the study, with population demographics described in Table 1. All the participants recorded a migraine day, and 14 of 16 participants recorded a non-migraine headache day. All participants had stable medications throughout the study period, and 50% were receiving botulinum toxin, 12.5% CGRP monoclonal antibodies and 25% oral preventative therapies in treatment of their migraines. 87.5% (14/16) of the cohort used triptans as an acute abortive agent, and the remainder non-steroidal anti-inflammatories.

**Table 1:** Population demographics, MHD; monthly headache days, MMD; Monthly migraine days, SD; standard deviation

	Population *N* = 16
Age Mean (SD)	45.7 (11.5)
Female *N* (%)	13 (81.3%)
Previous preventers Median (IQR)	4 (2)
Non-migraine MHD Mean (SD)	4 (3.3)
MMD Mean (SD)	6 (3.8)
Triptan use Number (%)	14 (87.5%)
NSAID use Number (%)	2 (12.5%)

During the headache phase of a migraine day, response in the SRT task was 8.2% slower than baseline (*z* =  − 2.923, *p* = 0.003), the average response time in the CRT was 10.2% slower (*z* =  − 3.206, *p* < 0.001), and response time in the WM was 5.6% slower (*z* =  − 2.172, *p* = 0.030). The accuracy of response for SRT (*z* =  − 2.219, *p* = 0.026), CRT (*z* =  − 2.097, *p* = 0.036) and WM tasks (*z* =  − 2.371, *p* = 0.018) were all significantly lower than baseline. A summary of test results is provided in Table 2.

**Table 2:** Median cohort results to MSReactor computerised cognitive battery in different headache states

	Interictal *N* = 16	Prodrome *N* = 16	Migraine *N* = 16	Postdrome *N* = 16	Non-migraine Headache *N* = 14
SRT (ms) Median (IQR)	322.478 (99.063)	310.815 (110.995)	349.582 (116.214)	316.12 (117.241)	296.484 (97.554)
CRT (ms) Median (IQR)	480.844 (120.58)	492.662 (102.509)	529.113 (133.416)	507.577 (131.641)	499.534 (94.633)
WM (ms) Median (IQR)	571.488 (83.617)	587.514 (156.443)	641.293 (150.728)	616.595 (160.667)	648.636 (138.817)
SRT percentage accuracy Median (IQR)	100 (1.5)	98.5 (3)	98.5 (3)	97 (3)	97 (4)
CRT percentage accuracy Median (IQR)	100 (2.6)	100 (0)	97 (7)	100 (3)	100 (7)
WM percentage accuracy Median (IQR)	97 (5.3)	97 (3)	94 (6)	94 (5)	97 (8)

*SRT* simple reaction test, *CRT* choice reaction test, *WM* working memory test, *IQR* inter-quartile range

**Fig. 1:**
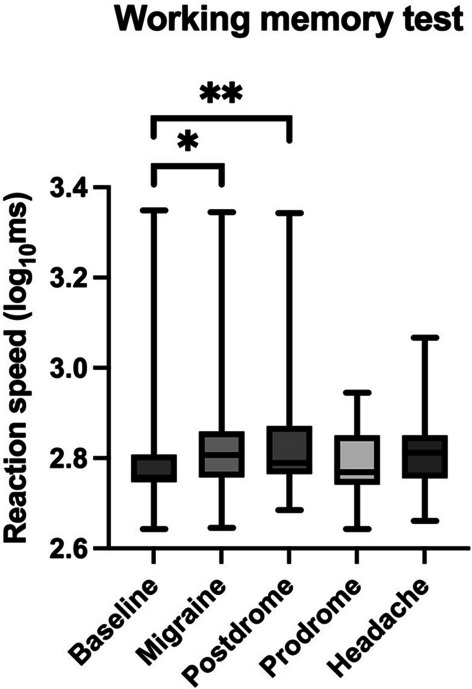
Box and whisker plot of reaction speed to working memory (WM) test in different migraine states expressed in log_10_ milliseconds, *p = 0.030 **p = 0.028

On the postdromal test day, the WM test was 8.9% slower than baseline (*z* =  − 2.198, *p* = 0.028), and significantly less accurate (*z* =  − 2.456, *p* = 0.014). Response speed for the WM test is summarised in Fig. 1. SRT on postdromal days was less accurate than baseline (*z* =  − 2.067, *p* = 0.039); however, response times for SRT and CRT were not significantly different. Neither the prodromal test day nor non-migraine headache test day were associated with a change in SRT, CRT or WM response time or accuracy compared to interictal baseline. There was no significant difference in severity of pain on migraine and non-migraine headache days (*z* =  − 0.051, *p* = 0.959).

## Discussion

We showed that performance on validated tests of WM, SRT and CRT worsens during the headache and postdromal phase of a migraine, but not on a non-migraine headache day, in comparison to a patients interictal baseline. This is the first study to utilise home-based computerised cognitive tests to examine the effect of cognition during the phases of a migraine, and a non-migrainous headache day.

These findings are broadly in keeping with several previous studies that have variably reported transient declines in processing speed, working memory and immediate and sustained attention during the headache phase of a migraine [7, 9]. It provides further validation of patient reports of cognitive symptoms during the migraine postdromal phase [2, 3].

Possible explanations for altered cognition in migraine have been summarised previously [13]. Previous observations include increased functional connectivity and fMRI activation in the temporal lobe in patients with migraine, and deficits in task-related suppression during acute pain, irrespective of pain catastrophizing or pain intensity [14–16]. Variations in grey matter density have also been reported in patients with migraine [17].

Our study provides several insights into contributory mechanisms to cognitive symptoms in migraine. Firstly, our observation of an impact on test scores on a migraine, but not on a non-migraine headache day, is significant. One hypothesis and possible confounder is that cognitive symptoms and test results relate to ‘distraction by pain’, our observation that there was no significant difference in pain severity between migraine and headache days, but that only migraine days were associated with significant deviations on testing, suggests that distraction by pain is not an adequate explanation for cognitive symptoms. Given deficits in task-related suppression during acute pain seen in fMRI studies however [15], pain remains a significant possible confounder in the design of cognitive study.

Secondly, the findings of our study describe a dynamic process impacting markers of cognition in migraine, that is not completely explained by static changes in grey matter density. In Mathur et al.’s fMRI study, patients with migraine underwent cognitive testing during and in the absence of painful stimuli [16]. Evidence of abnormally blunted cognitive task-related deactivation of the left dorsolateral prefrontal cortex and left dorsal anterior midcingulate cortex suggested alterations of cognitive processing in migraine, which were further modulated by migraine frequency [16]. Furthermore, functional studies have also shown increased activation of cortical areas related to executive function during a migraine attack [18]. Taken together, this suggests maladaptive functional connectivity of altered pain-cognition networks in migraine that are associated with attack frequency, which may explain the association of migraine with cognitive symptoms.

There are several limitations to this study. The lack of observed change during the defined prodromal period may relate to the timing of the test, which was defined retrospectively from the headache phase rather than prospectively from reported symptoms. Secondly, due to the study design and sample size, the generalisability to chronic migraine, and association with disease frequency and duration were not investigated, however the study design, utilising paired samples, controls for variation in education and background. The study population included patients who were on a preventative agent for migraine. As they were all on a stable regimen, this was felt to not be responsible for the observed variation in cognition, and an accurate representation of the lived experience of a patient with migraine. Finally, the effect of other variables such as altered sleep during a migraine was not examined. Given the observation that poor sleep quality may precede a migraine attack [19, 20] and the impact of sleep on cognitive performance [21], this is a significant confounder that requires further study.

## Conclusion

Cognitive test performance in terms of working memory, simple reaction and choice reaction time is lower on a migraine and postdromal day compared to interictally in patients with episodic migraine. Further study is required to assess the interaction between migraine frequency, severity, disease duration and sleep on cognitive function. Sensitive cognitive testing may allow migraineurs to determine when they should return to work duties that depend on their normal cognitive function.

## Data Availability

Primary data is available on reasonable request to the corresponding author.
